# Sexual quality of life among patients with lung cancer and people living with HIV: a cross-sectional study

**DOI:** 10.3389/fpsyg.2026.1766006

**Published:** 2026-03-02

**Authors:** Marta Milewska-Buzun, Anna Baranowska, Rafal Buzun, Mateusz Zarychta, Mateusz Cybulski

**Affiliations:** 1 Department of Integrated Medical Care, Faculty of Health Sciences, Medical University of Bialystok, Bialystok, Poland; 2 Faculty of Mechanical Engineering and Aeronautics, Rzeszow University of Technology, Rzeszow, Poland; 3 Faculty of Technical Physics, Information Technology and Applied Mathematics, Lodz University of Technology, Lodz, Poland

**Keywords:** human immunodeficiency virus, lung cancer, sexual desire, sexual function, sexual quality of life, sexual satisfaction

## Abstract

**Introduction:**

Sexual quality of life is an important component of health and wellbeing for people with chronic diseases.

**Objective:**

The aim of the study was to assess sexual quality of life among patients with lung cancer and people living with HIV.

**Methods:**

The study included 191 participants (98 men and 93 women) diagnosed with lung cancer or HIV infection. The study used a proprietary questionnaire, the Female Sexual Function Index (FSFI), the International Index of Erectile Function (IIEF-15), and the Sexual Quality of Life—Female (SQoL-F) and Male (SQoL-M).

**Results:**

Among men, statistically significant negative relationships were observed between age and erectile function (*p* = 0.04), orgasmic function (*p* = 0.018), and sexual desire (*p* = 0.032) on the IIEF-15. Sexual desire among women was significantly negatively correlated with age in the lung cancer group (*p* = 0.007). The level of formal education had a significant influence on the scores in individual domains on the IIEF-15. The analysis indicated a significant relationship of formal education level on sexual quality of life in both men and women. The analysis performed using the Kruskal-Wallis test revealed significant differences in erectile function (*p* = 0.044) and orgasmic function (*p* = 0.012) in men depending on financial status. In terms of SQoL-F, women who rated their financial situation as “neither good nor bad” scored significantly lower than women with a good financial situation (*p* = 0.007).

**Discussion:**

In conclusion, sociodemographic factors such as age, formal education, and financial status, significantly influenced the functioning of patients living with HIV and those diagnosed with lung cancer. Individuals living with HIV demonstrated higher levels of sexual functioning and sexual quality of life compared with patients with lung cancer. A better financial situation, and higher formal educational attainment were associated with higher scores on scales used in the study.

## 1 Introduction

The quality of sexual life is a significant element of human health, influencing mental wellbeing, interpersonal relationships, and self-esteem (Cranney, 2017). It is closely related to overall life satisfaction as well as to the emotional and social functioning of the individuals (Shahrahmani et al., 2024; Eden and Wylie, 2009). Many chronic diseases and the treatments applied in their course lead to sexual dysfunctions, which may negatively affect adherence, self-esteem, and interpersonal relationships. Nevertheless, the topic of sexual quality of life rarely arises in clinical discussions. This is likely due to the assumption that sexual health is not a priority in the management of the underlying disease (Flynn et al., 2016).

In the present study, a deliberate comparison was made between two groups of patients: individuals living with HIV and patients diagnosed with lung cancer. The introduction of effective antiretroviral therapy (ART) has significantly improved the prognosis of people living with HIV; however, it has also led to an increase in the incidence of non-AIDS-defining cancers (NADCs), including lung cancer, which is now among the most frequently diagnosed malignancies in this population. Compared to individuals without HIV infection, patients living with HIV develop lung cancer at a younger age (on average at 50 years), which corresponds to the period of life when individuals are typically sexually active (Hakimian et al., 2007; Shiels et al., 2011; Sigel et al., 2012; Stewart et al., 2012; Ehren et al., 2014; Reekie et al., 2010; Worm et al., 2013).

Patients in both groups experience significant psychological burdens associated with diagnosis, treatment, prognosis, and anxiety about the future. In both cases, social stigmatization is present, although its sources and nature differ. In HIV, stigmatization is linked to negative stereotypes regarding modes of transmission and fear of infecting a partner, which affects self-perception of sexuality, feelings of guilt, and fear of rejection (Reese and Haythornthwaite, 2016; Erlandson and Karris, 2019). In lung cancer, stigmatization primarily stems from the association of the disease with tobacco smoking and from social distancing toward patients, which in-fluences body image and social interactions (Oberguggenberger et al., 2024).

The impact of illness on sexual life differs between the two groups. In HIV, sexuality is directly linked to the disease itself—through the risk of viral transmission, fear of infecting a partner, and restrictions on intimate contact (Reese and Haythornthwaite, 2016; Erlandson and Karris, 2019). In lung cancer, sexual life is primarily limited by reduced physical condition, disease symptoms, and treatment-related side effects (e.g., fatigue, dyspnea, hormonal disturbances) (Maruelli et al., 2014; Aptecar et al., 2021). Despite these differences, patients in both groups experience similar emotional con-sequences, including decreased libido, diminished sense of attractiveness, difficulties in maintaining relationships, and fear of rejection by a partner (Aptecar et al., 2021; Morrison et al., 2017).

The comparison of these two groups—despite differences in disease etiology and clinical course—thus allows for an assessment of the impact of distinct pathological and psychosocial mechanisms on sexual quality of life, as well as for the identification of factors that are shared or specific to each population.

The aim of the study was to assess the sexual quality of life in both patient groups and to identify sociodemographic factors influencing sexual functioning among individuals diagnosed with lung cancer and those living with HIV. The following research question was formulated:

*Q1*: Are there differences in sexual quality of life between patients living with HIV and those diagnosed with lung cancer?

*Q2*: Do sociodemographic variables influence sexual function in people living with HIV and lung cancer patients?

*Q3*: What sociodemographic factors are associated with sexual quality of life among patients living with HIV and those diagnosed with lung cancer?

## 2 Materials and methods

### 2.1 Study design

All study participants were patients of the University Teaching Hospital in Bialystok, admitted to the Department of Infectious Diseases and Hepatology with Subunit for HIV/AIDS Patients, the 1st Department of Pulmonary Diseases, Lung Cancer and Internal Medicine, and the 2nd Department of Pulmonary Diseases, Lung Cancer and Internal Medicine, as well as patients of the Counseling and Diagnostic Centre for adults with HIV and AIDS. The study was conducted between February 2024 and December 2024. None of the study participants suffered from two diseases at the same time.

### 2.2 Sample size

**Figure 1:**
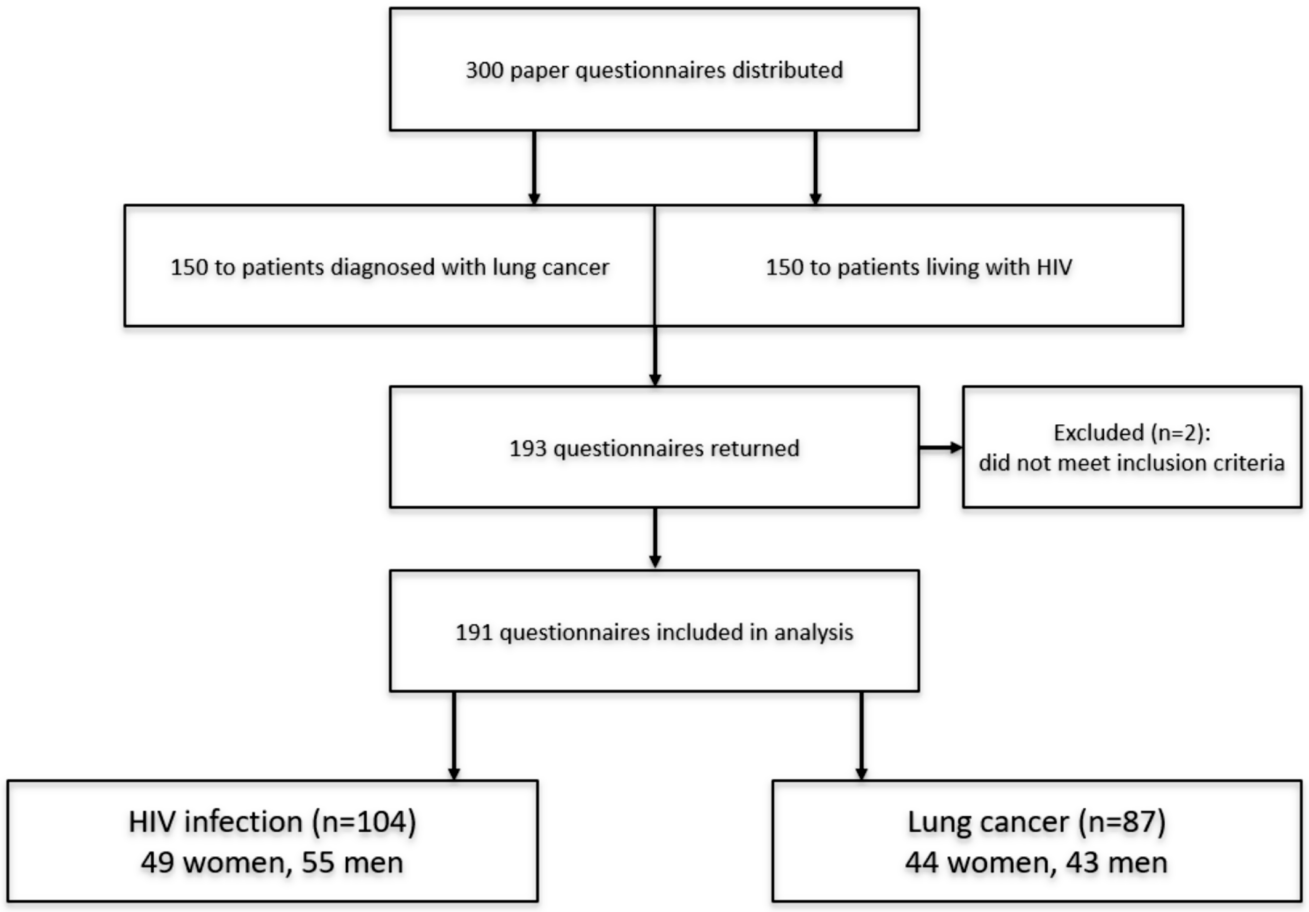
CONSORT flow diagram of study selection.

A total of 300 paper questionnaires were distributed as part of the study, 150 of which were given to patients diagnosed with lung cancer and 150 to people living with HIV. Of the total number of questionnaires distributed, 193 were returned, resulting in a response rate of 64.3%. Of the questionnaires returned, 191 were selected for further analysis, including 93 women (48.7%) and 98 men (51.3%). Among the participants, 104 were HIV patients (55 men and 49 women), and 87 were individuals diagnosed with lung cancer (43 men and 44 women). Two questionnaires were excluded from the analysis due to non-compliance with the study criteria: respondents reported having both diseases, whereas the study design only permitted participants with one of them (Figure 1).

### 2.3 Sampling technique

Participants were recruited using a convenience sampling approach from the above clinical settings. The questionnaires were distributed in person by researchers or medical staff, who provided participants with detailed instructions on how to fill them out and encouraged them to ask questions to address any unclear points.

### 2.4 Criteria

The inclusion criteria were a confirmed diagnosis of one of two diseases: lung cancer or HIV infection, as well as a minimum age of 18 years. Each respondent could withdraw from the study at any time.

### 2.5 Data collection

#### 2.5.1 Original questionnaire

The original questionnaire consisted of 15 questions, including eight closed-ended questions that covered sociodemographic characteristics such as sex, age, marital status, formal education level, place of residence, financial situation, employment status, and medical diagnosis. In addition, seven open-ended questions were included, concerning, among other things, self-assessment of sexual attractiveness, subjective assessment of health status, duration of illness, current treatment, relationships, concerns related to the disease, and their impact on sex life.

#### 2.5.2 The female sexual function index

The Female Sexual Function Index (FSFI) is a psychometric tool designed to assess various aspects of female sexual function. This tool helps identify difficulties in areas such as desire, arousal, lubrication, ability to orgasm, overall sexual satisfaction, and the presence of pain during sexual activity (Janus and Szulc, 2015; Wylomanski et al., 2014). The questionnaire contains 19 items grouped into six main thematic domains. The score for each of these categories is calculated by adding up the points assigned to specific questions within a given domain, ranging from 0 to 6 points. The higher the score, the better the sexual function in that area. Scores of 3.9 or lower in any domain suggest a clinically significant sexual dysfunction (Wiegel et al., 2005; Meston, 2003). Additionally, the total score for the entire questionnaire is calculated, ranging from 2 to 36 points (Wiegel et al., 2005; Meston, 2003). The questionnaire can be used in surveys among women at different stages of their sex lives, both before and after menopause (Meston, 2003). The FSFI has high internal consistency, with a Cronbach’s alpha of 0.97 overall (Rosen et al., 2000). Cronbach’s alpha in this study reached a value of 0.89, which also indicates high internal consistency of the FSFI. The Polish version of the FSFI scale was validated by Nowosielski et al. (2013).

#### 2.5.3 The sexual quality of life—female questionnaire

The Sexual Quality of Life—Female (SQoL-F) questionnaire was designed to assess the sexual quality of women’s lives. The validation study showed high internal consistency, with a Cronbach’s alpha of 0.93 (Symonds et al., 2005). Cronbach’s alpha in this study reached a value of 0.82, which also indicates high internal consistency of the SQoL-F. This tool contains 18 statements relating to emotions, thoughts, and feelings experienced by women in the context of their sexuality. The questions cover aspects such as perception of self-perceived attractiveness, intimate relationships with a partner, and overall emotional wellbeing related to sex life and relationships (Symonds et al., 2005). Respondents provided answers by selecting one of six options ranging from strong agreement to strong disagreement, which are scored on a scale from 6 to 0. The questionnaire score is calculated based on the answer key and then standardized to determine the level of sexual satisfaction. The scale ranges from 0 (complete dissatisfaction) to 100 points (complete satisfaction with sexual life). Higher total scores indicate a better subjective perception of sexual quality of life by the respondent (Symonds et al., 2005).

#### 2.5.4 The international index of erectile function (IIEF-15)

The International Index of Erectile Function is a multidimensional assessment tool for erectile dysfunction commonly used in clinical research and clinical practice. It is a standardized questionnaire with good sensitivity and specificity in identifying changes in sexual function, including those in response to therapeutic interventions and changes in clinical status. The IIEF-15 questionnaire comprises 15 items, with total scores ranging from 5 to 75 (Rosen et al., 1997). Five basic domains of sexual function are assessed: erectile function, intercourse satisfaction, orgasmic function, sexual desire, and overall satisfaction. The scores in individual domains are summed, with lower scores reflecting greater severity of dysfunction. The overall sexual activity assessed in the erectile function domain below 6 points in the past 4 weeks may indicate negligible sexual activity, thus preventing a reliable diagnosis of erectile dysfunction using the IIEF-15 (Rosen et al., 1997). The criterion for diagnosing erectile dysfunction is a score of ≤25 in the erectile function domain. The severity of dysfunction in this domain is classified as follows:

No erectile dysfunction: score 26–30,
Mild erectile dysfunction: score 22–25,
Mild to moderate erectile dysfunction: score 17–21,
moderate erectile dysfunction: score 11–16,
severe erectile dysfunction: score 6–10 (Rosen et al., 1997).

This tool has excellent psychometric reliability, with a Cronbach’s alpha of 0.91 overall (Rosen et al., 1997). Cronbach’s alpha in this study reached a value of 0.97, which also indicates a very high internal consistency of the IIEF-15.

#### 2.5.5 The sexual quality of life—male questionnaire

The SQoL-M questionnaire is used to assess the impact of sexual dysfunction on men’s quality of life, including their self-esteem, relationships, and emotional state. It is a modified version of the SQoL-F tool used in women.

It consists of 11 statements and is structured on a six-point Likert scale, allowing respondents to rate the extent to which they agree with the statements. As an interval and bipolar scale, it captures both the polarity and intensity of the beliefs expressed (1 = strongly agree—the lowest quality of life, 6 = strongly disagree—the highest quality of life). The results are converted to a standardized scale of 0–100, where higher scores indicate better sexual quality of life (Abraham et al., 2008). The questionnaire demonstrates high internal consistency, with a Cronbach’s alpha of ≥0.82 across all study groups (Abraham et al., 2008). Cronbach’s alpha in this study reached a value of 0.91, which also indicates a very high internal consistency of the SQoL-M.

Because the study involved potentially vulnerable patient groups and sensitive questions, participation was strictly voluntary and based on written informed consent. The survey was anonymous and completed under conditions ensuring privacy and freedom of response. Participants could refuse to answer any item and withdraw at any time without consequences for their medical care. The protocol complied with the Declaration of Helsinki and was approved by the Bioethics Committee of the Medical University of Bialystok (resolution No. APK.002.142.2024 of 22 February 2024).

### 2.6 Data analysis

Data were statistically analyzed using the Statistica 13.3 package (StatSoft Poland, Krakow, Poland). The normality of quantitative variables was determined using the Shapiro–Wilk test. Because none of the analyzed variables were found to be normally distributed, non-parametric tests were employed. The significance of differences between the two groups was assessed using the Mann–Whitney U test, and for comparisons among multiple groups, the Kruskal–Wallis test and its corresponding *post hoc* tests were employed. The significance of relationships between qualitative variables was verified using Pearson’s chi-squared test (χ^2^). The relationships between quantitative variables were analyzed using Spearman’s rank correlation coefficient. All test results were considered statistically significant at *p* < 0.05.

## 3 Results

### 3.1 Sociodemographic characteristics of the respondents

The average age of the respondents was 56 years, with men being older than women (61 vs. 50). The mean duration of illness was 4 years and did not differ significantly between the sexes (*p* = 0.795). Tables 1, 2 present detailed sociodemographic data of the respondents, including information on disease duration and sexual orientation.

**Table 1:** Age and duration of illness of the respondents.

Variable	Group	*M*	SD	Me	*p*
Age	Men (*n* = 98)	61.10	11.39	63.50	<0.001*
	Women (*n* = 93)	50.74	17.45	60.00	
	Total (*n* = 191)	56.06	15.51	61.00	
Duration of illness (years)	Men (*n* = 98)	4.70	6.14	2.00	0.795
	Women (*n* = 93)	3.93	4.97	2.00	
	Total (*n* = 191)	4.33	5.60	2.00	

M, mean; Me, median; SD, standard deviation; p, *p*-value; *statistically significant value.

**Table 2:** Sociodemographic data of the respondents.

Variable		HIV		Lung cancer		Total		*p*
		*n*	%	*n*	%	*n*	%	
Sex	Male	40	48.19	58	53.70	98	51.31	0.450
	Female	43	51.81	50	46.30	93	48.69	
Education	Primary	7	8.4	18	17.0	25	13.1%	0.003*
	Vocational	22	26.5	44	41.5	66	34.6%	
	Secondary	40	48.2	25	23.6	65	34.0%	
	Tertiary	14	16.9	19	17.9	33	17.3%	
Place of residence	Rural area	26	31.33	27	25.00	53	27.7%	0.830
	Town/city up to 50,000 inhabitants	20	24.10	30	27.78	50	26.2%	
	Town/city with 50–100,000 inhabitants	10	12.05	12	11.11	22	11.5%	
	Town/City with 100,000–200,000 inhabitants	8	9.64	9	8.33	17	8.9%	
	Town/City with over 200,000 inhabitants	19	22.89	30	27.78	49	25.7%	
Marital status	Single	40	48.19	3	2.78	43	22.5%	<0.001*
	Married	20	24.10	64	59.26	84	44.0%	
	Divorced	20	24.10	16	14.81	36	18.8%	
	Widowed	3	3.61	24	22.22	27	14.1%	
Relationship status	Formal relationship	18	21.69	65	60.19	83	43.5%	<0.001*
	Informal relationship	21	25.3	16	14.81	37	19.4%	
	Unmarried	44	53.01	27	25.00	71	37.2%	
Sexual orientation	Homosexual	12	14.46	13	12.04	25	13.1%	0.002*
	Heterosexual	60	72.29	92	85.19	152	79.6%	
	Bisexual	11	13.25	1	0.93	12	6.3%	
Employment status**	Student	11	13.25	2	1.85	13	6.8%	0.004*
	Employed	51	61.45	26	24.07	77	40.3%	<0.001*
	Disability pension	14	16.87	10	9.26	24	12.6%	0.116
	Retirement pension	3	3.61	66	61.11	69	36.1%	<0.001*
	Unemployed	5	6.02	4	3.70	10	5.2%	0.453
Financial situation	Very bad	0	0	0	0	0	0.0%	0.004*
	Bad	15	18.07	5	4.63	20	10.5%	
	Neither good nor bad	34	40.96	36	33.33	70	36.6%	
	Good	29	34.94	61	56.48	90	47.1%	
	Very good	5	6.02	6	5.56	11	5.8%	

p, *p*-value; *statistically significant value; **more than one answer could be selected, % do not add up to 100%.

Significant differences were observed in the level of formal education (*p* = 0.003). Individuals living with HIV were more likely to have completed secondary education (48.2%), whereas patients with lung cancer more often reported vocational education (41.5%). Substantial differences were also found regarding marital status (*p* < 0.001) and relationship status (*p* < 0.001). In the HIV group, single individuals (48.2%) and those not currently in a relationship (53.0%) predominated, while among lung cancer patients, the majority were in formal relationships (60.2%) or married (59.3%). With respect to sexual orientation, most respondents identified as heterosexual (79.6%); however, homosexual (14.5%) and bisexual (13.3%) individuals were more common in the HIV group than among lung cancer patients (12.0 and 0.9%, respectively). Clear differences were also found in employment status (*p* < 0.001). In the HIV group, the majority were employed (61.5%), whereas retirees predominated among lung cancer patients (61.1%). Differences were additionally noted in the assessment of financial situation (*p* = 0.004): patients with lung cancer more frequently rated their financial situation as good or very good (62.0%), while individuals living with HIV more often described it as average or poor.

### 3.2 Comparative analysis of sexual function and sexual quality of life in patients with HIV and lung cancer

In male patients living with HIV, significantly higher scores were observed in the erectile function domain (*p* = 0.020) and sexual desire domain (*p* < 0.001) compared to men with lung cancer. In the other domains of the IIEF-15, the differences were not statistically significant. The mean score of male sexual quality of life (SQoL-M) was slightly higher among people living with HIV, but the difference was not statistically significant (*p* = 0.144).

A similar relationship was observed among women. Female patients with HIV scored significantly higher on the FSFI desire domain (*p* = 0.002) compared to those with lung cancer. In terms of female sexual quality of life (SQoL-F), patients with lung cancer achieved higher mean scores than those with HIV (*p* = 0.054), although this result was bordering on statistical significance (Table 3).

**Table 3:** Sexual function and sexual quality of life in patients with HIV and lung cancer.

Scale	HIV					Lung cancer			*p*
	*n*	*M*	SD	Me	*n*	*M*	SD	Me	
IIEF-15 erectile function	40	11.0	10.71	4.00	58	7.69	9.28	3.00	0.020*
IIEF-15 orgasmic function	40	3.42	4.33	0.00	58	2.22	3.64	0.00	0.168
IIEF-15 sexual desire	40	5.65	2.35	6.00	58	3.74	2.35	3.00	<0.001*
IIEF-15 intercourse satisfaction	40	4.18	5.80	0.00	58	2.90	5.04	0.00	0.423
IIEF-15 overall satisfaction	40	5.78	2.54	6.00	58	4.95	2.30	6.00	0.110
FSFI desire	43	3.92	1.65	3.60	50	2.83	1.29	3.00	0.002*
FSFI arousal	43	2.55	2.44	2.70	50	2.65	1.74	2.55	0.635
FSFI lubrication	43	3.15	2.57	4.20	50	3.26	2.12	3.60	0.901
FSFI orgasm	43	2.91	2.41	4.00	50	3.02	2.02	3.60	0.919
FSFI satisfaction	43	3.62	2.11	4.80	50	3.69	1.31	3.60	0.709
FSFI pain	43	4.33	2.34	6.00	50	4.34	2.02	5.20	0.604
Overall FSFI	43	20.49	10.62	26.90	50	19.79	8.98	20.70	0.694
SQoL-M	40	0.66	0.30	0.75	58	0.62	0.23	0.67	0.144
SQoL-F	43	0.50	0.31	0.35	50	0.62	0.23	0.62	0.054

FSFI, Female Sexual Function Index; IIEF-15, International Index of Erectile Function; M, mean; Me, median; SD, standard deviation; SQoL-F, Sexual Quality of Life, Female; SQoL-M, Sexual Quality of Life, Male; p, *p*-value; *statistically significant value.

### 3.3 The relationship of sociodemographic variables on sexual quality of life

#### 3.3.1 Correlations between age and sexual function and sexual quality of life

Table 4 presents an analysis of the relationship between age and scores on three scales evaluating sexual function: IIEF-15 (for men), FSFI (for women), and SQoL (for both sexes).

**Table 4:** Correlation coefficients between scale scores and age.

Sex (disease)	Scale	*n*	*r*	*p*
Men (HIV)	IIEF-15 Erectile function	40	−0.440	0.04*
	IIEF-15 Orgasmic function	40	−0.372	0.018*
	IIEF-15 Sexual desire	40	−0.339	0.032*
	IIEF-15 Intercourse satisfaction	40	−0.167	0.302
	IIEF-15 Overall satisfaction	40	−0.206	0.201
Women (HIV)	FSFI Desire	43	−0.107	0.495
	FSFI Arousal	43	0.093	0.552
	FSFI Lubrication	43	0.040	0.798
	FSFI Orgasm	43	0.074	0.635
	FSFI Satisfaction	43	−0.025	0.875
	FSFI Pain	43	−0.087	0.578
	Overall FSFI	43	−0.011	0.944
Men (lung cancer)	IIEF-15 Erectile function	58	−0.317	0.015*
	IIEF-15 Orgasmic function	58	−0.181	0.175
	IIEF-15 Sexual desire	58	−0.191	0.150
	IIEF-15 Intercourse satisfaction	58	−0.185	0.164
	IIEF-15 Overall satisfaction	58	−0.423	0.001*
Women (lung cancer)	FSFI Desire	50	−0.375	0.007*
	FSFI Arousal	50	−0.335	0.017*
	FSFI Lubrication	50	−0.314	0.026*
	FSFI Orgasm	50	−0.172	0.232
	FSFI Satisfaction	50	−0.136	0.346
	FSFI Pain	50	−0.253	0.076
	Overall FSFI	50	−0.295	0.038*
Men (HIV)	SQoL	40	−0.317	0.046*
Women (HIV)		43	0.357	0.019*
Total (HIV)		83	0.130	0.241
Men (lung cancer)	SQoL	58	−0.101	0.452
Women (lung cancer)		50	−0.007	0.964
Total (lung cancer)		108	0.085	0.383

FSFI, Female Sexual Function Index; IIEF-15, International Index of Erectile Function; SQoL-F, Sexual Quality of Life, Female; SQoL-M, Sexual Quality of Life, Male; r, Spearman’s rank correlation coefficient; p, *p*-value; *statistically significant value.

In men, statistically significant negative relationships were observed between age and the following domains: erectile function (*p* = 0.04), orgasmic function (*p* = 0.018), and sexual desire (*p* = 0.032) on the IIEF-15. In men with lung cancer, a negative relationship was observed with overall sexual satisfaction (*p* = 0.001) as measured by the IIEF-15 scale. For the sexual quality of life (SQoL-M), the correlation with age was negative but not statistically significant (*p* = 0.075).

Sexual desire among women was significantly negatively correlated with age in the lung cancer group (*p* = 0.007) and non-significantly in the HIV group. Among women with lung cancer, age was also negatively correlated with arousal (*p* = 0.017), lubrication (*p* = 0.026), and the overall FSFI score (*p* = 0.038). In contrast, among women living with HIV, age was positively correlated with sexual quality of life (*p* = 0.019). Analysis of SQoL by gender revealed no significant correlations with age in either the HIV group or the lung cancer group.

#### 3.3.2 The level of formal education, marital status, relationship status, and financial situation and sexual function

The analysis revealed that the level of formal education had a significant influence on the scores in individual domains on the IIEF-15 (Table 5). The lowest scores were recorded among men with vocational education, while the highest scores were recorded among those with secondary education. Significant differences between groups were noted in the following domains: erectile function (*p* = 0.021), orgasmic function (*p* = 0.036), sexual desire (*p* < 0.001), intercourse satisfaction (*p* < 0.001), and overall satisfaction (*p* < 0.001). In each domain, vocational education was correlated with lower levels of sexual function compared to secondary and tertiary education.

**Table 5:** The level of formal education, marital status, relationship status and financial situation and the scores on individual scales.

Scale	Variable		*n*	*M*	SD	Me	K-W	*p* *Post hoc*
IIEF-15 Erectile function	Education	Primary (I)	20	9.30	9.47	3.50	0.021*	I–II NS I–III NS I–IV NS II–III < 0.001* II–IV NS III–IV NS
		Vocational (II)	45	6.09	7.78	3.00		
		Secondary (III)	18	17.17	11.69	24.00		
		Tertiary (IV)	13	8.85	10.15	3.00		
	Marital status	Single (I)	18	9.72	10.35	4.00	0.136	-
		Married (II)	52	10.21	10.43	3.00		
		Divorced (III)	17	9.00	10.35	4.00		
		Widowed (IV)	11	2.45	0.52	2.00		
	Financial situation	Bad (I)	9	5.11	7.90	2.00	0.044*	I–II NS I–III NS I–IV 0.049* II–III NS II–IV NS III–IV NS
		Neither good nor bad (II)	34	7.82	9.97	3.00		
		Good (III)	51	9.29	9.61	4.00		
		Very good (IV)	4	25.00	3.27	25.00		
IIEF-15 Orgasmic function	Education	Primary (I)	20	2.90	3.93	0.00	0.017*	I–II NS I–III NS I–IV NS II–III 0.036* II–IV NS III–IV NS
		Vocational (II)	45	1.47	2.93	0.00		
		Secondary (III)	18	5.50	4.63	8.00		
		Tertiary (IV)	13	3.31	4.61	0.00		
	Marital status	Single (I)	18	3.22	4.39	0.00	0.057	-
		Married (II)	52	3.23	4.04	0.00		
		Divorced (III)	17	2.35	4.01	0.00		
		Widowed (IV)	11	0.00	0.00	0.00		
	Financial situation	Bad (I)	9	1.11	3.33	0.00	0.012*	I–II NS I–III NS I–IV 0.036* II–III NS II–IV NS III–IV NS
		Neither good nor bad (II)	34	2.26	3.78	0.00		
		Good (III)	51	2.80	3.91	0.00		
		Very good (IV)	4	9.00	1.15	9.00		
IIEF-15 Sexual desire	Education	Primary (I)	20	4.45	2.37	4.00	<0.001*	I–II NS I–III NS I–IV NS II–III < 0.001* II–IV NS III–IV NS
		Vocational (II)	45	3.80	2.32	2.00		
		Secondary (III)	18	6.67	2.52	8.00		
		Tertiary (IV)	13	4.54	1.94	4.00		
	Marital status	Single (I)	18	6.22	1.93	6.00	<0.001*	I–II 0.012* I–III NS I–IV 0.002* II–III NS II–IV NS III–IV NS
		Married (II)	52	4.21	2.55	3.00		
		Divorced (III)	17	4.88	2.67	4.00		
		Widowed (IV)	11	2.64	0.92	2.00		
	Financial situation	Bad (I)	9	3.56	1.81	3.00	0.125	-
		Neither good nor bad (II)	34	4.47	2.54	4.00		
		Good (III)	51	4.51	2.60	3.00		
		Very good (IV)	4	7.25	0.96	7.50		
IIEF-15 Intercourse satisfaction	Education	Primary (I)	20	4.00	5.77	0.00	<0.001*	I–II NS I–III NS I–IV NS II–III 0.005* II–IV NS III–IV NS
		Vocational (II)	45	1.67	3.90	0.00		
		Secondary (III)	18	7.78	6.52	11.50		
		Tertiary (IV)	13	3.08	4.65	1.00		
	Marital status	Single (I)	18	2.89	5.59	0.00	0.569	-
		Married (II)	52	4.35	5.66	0.00		
		Divorced (III)	17	3.12	5.42	0.00		
		Widowed (IV)	11	0.36	0.50	0.00		
	Financial situation	Bad (I)	9	1.33	4.00	0.00	0.052	-
		Neither good nor bad (II)	34	2.65	4.99	0.00		
		Good (III)	51	3.86	5.55	0.00		
		Very good (IV)	4	9.00	6.00	12.00		
IIEF-15 Overall satisfaction	Education	Primary (I)	20	6.35	1.04	6.00	<0.001*	I–II 0.004* I–III NS I–IV NS II–III < 0.001* II–IV 0.002* III–IV NS
		Vocational (II)	45	3.98	2.23	3.00		
		Secondary (III)	18	6.72	2.56	7.50		
		Tertiary (IV)	13	6.69	1.65	7.00		
	Marital status	Single (I)	18	4.89	2.76	5.00	0.535	-
		Married (II)	52	5.63	2.42	6.00		
		Divorced (III)	17	4.88	2.12	5.00		
		Widowed (IV)	11	4.91	2.34	6.00		
	Financial situation	Bad (I)	9	5.11	2.20	6.00	0.107	-
		Neither good nor bad (II)	34	4.74	2.44	5.50		
		Good (III)	51	5.51	2.41	6.00		
		Very good (IV)	4	7.50	1.73	8.00		
FSFI Desire	Education	Primary (I)	5	2.16	1.38	1.20	0.164	-
		Vocational (II)	21	3.09	1.17	3.60		
		Secondary (III)	47	3.36	1.86	2.40		
		Tertiary (IV)	20	3.84	0.92	3.60		
	Marital status	Single (I)	25	3.79	1.89	3.60	<0.001*	I–II NS I–III NS I–IV 0.002* II–III 0.002* II–IV NS III–IV < 0.001*
		Married (II)	32	2.96	1.01	3.00		
		Divorced (III)	19	4.58	0.96	4.20		
		Widowed (IV)	16	1.91	1.10	1.20		
	Relationship status	Formal (I)	32	2.89	1.05	3.00	0.011*	I–II 0.011* I–III NS II–III NS
		Informal (II)	21	4.14	1.02	4.20		
		None (III)	40	3.27	1.95	3.00		
	Financial situation	Bad (I)	11	2.73	2.22	1.20	0.246	–
		Neither good nor bad (II)	36	3.68	1.57	3.60		
		Good (III)	39	3.25	1.42	3.60		
		Very good (IV)	7	3.00	0.69	3.00		
FSFI Arousal	Education	Primary (I)	5	1.98	1.96	2.40	0.029*	I–II NS I–III NS I–IV NS II–III NS II–IV NS III–IV 0.030*
		Vocational (II)	21	2.36	1.63	2.40		
		Secondary (III)	47	2.23	2.14	2.10		
		Tertiary (IV)	20	3.89	2.01	4.35		
	Marital status	Single (I)	25	2.21	2.36	1.50	0.199	-
		Married (II)	32	3.15	1.69	3.15		
		Divorced (III)	19	2.65	2.46	3.90		
		Widowed (IV)	16	1.95	1.69	1.80		
	Relationship status	Formal (I)	32	3.10	1.69	3.00	<0.001*	I–II NS I–III 0.013* II–III 0.003*
		Informal (II)	21	3.56	2.13	4.20		
		None (III)	40	1.70	2.02	1.20		
	Financial situation	Bad (I)	11	0.52	1.52	0.00	<0.001*	I–II 0.041* I–III 0.004* I–IV 0.002* II–III NS II–IV NS III–IV NS
		Neither good nor bad (II)	36	2.47	1.91	2.25		
		Good (III)	39	3.04	2.14	3.00		
		Very good (IV)	7	4.16	0.44	4.50		
FSFI Lubrication	Education	Primary (I)	5	2.58	2.45	3.60	0.016*	I–II NS I–III NS I–IV NS II–III NS II–IV NS III–IV 0.013*
		Vocational (II)	21	3.44	1.76	3.90		
		Secondary (III)	47	2.69	2.45	3.00		
		Tertiary (IV)	20	4.37	2.23	5.40		
	Marital status	Single (I)	25	3.28	2.60	4.50	0.340	-
		Married (II)	32	3.78	1.83	3.90		
		Divorced (III)	19	2.84	2.59	4.20		
		Widowed (IV)	16	2.29	2.32	2.10		
	Relationship status	Formal (I)	32	3.76	1.83	3.90	0.072	-
		Informal (II)	21	3.81	2.27	4.50		
		None (III)	40	2.46	2.54	2.10		
	Financial situation	Bad (I)	11	0.49	1.63	0.00	<0.001*	I–II 0.045* I–III < 0.001* I–IV < 0.001* II–III NS II–IV 0.023* III–IV NS
		Neither good nor bad (II)	36	2.94	2.12	3.60		
		Good (III)	39	3.82	2.15	4.20		
		Very good (IV)	7	5.53	0.62	5.70		
FSFI Orgasm	Education	Primary (I)	5	2.32	2.29	2.80	0.182	-
		Vocational (II)	21	3.49	1.99	4.00		
		Secondary (III)	47	2.53	2.33	3.20		
		Tertiary (IV)	20	3.62	1.91	4.00		
	Marital status	Single (I)	25	3.06	2.50	3.60	0.311	-
		Married (II)	32	3.49	1.62	3.60		
		Divorced (III)	19	2.61	2.44	3.20		
		Widowed (IV)	16	2.08	2.26	1.60		
	Relationship status	Formal (I)	32	3.41	1.60	3.60	0.051	-
		Informal (II)	21	3.64	2.17	4.40		
		None (III)	40	2.26	2.46	1.60		
	Financial situation	Bad (I)	11	0.29	0.96	0.00	<0.001*	I–II 0.011* I–III < 0.001* I–IV 0.002* II–III NS II–IV NS III–IV NS
		Neither good nor bad (II)	36	2.83	2.19	3.40		
		Good (III)	39	3.59	2.03	4.00		
		Very good (IV)	7	4.40	0.61	4.40		
FSFI Satisfaction	Education	Primary (I)	5	3.44	1.12	3.20	0.446	-
		Vocational (II)	21	4.00	1.51	4.80		
		Secondary (III)	47	3.37	1.89	3.60		
		Tertiary (IV)	20	4.02	1.58	4.80		
	Marital status	Single (I)	25	3.63	2.26	3.60	0.201	-
		Married (II)	32	4.14	1.10	4.20		
		Divorced (III)	19	3.39	1.88	4.40		
		Widowed (IV)	16	3.00	1.46	2.80		
	Relationship status	Formal (I)	32	4.09	1.11	4.00	<0.001*	I–II NS I–III 0.031* II–III 0.002*
		Informal (II)	21	4.46	1.67	4.80		
		None (III)	40	2.89	1.86	2.60		
	Financial situation	Bad (I)	11	1.93	1.25	1.20	0.001*	I–II NS I–III 0.006* I–IV 0.002* II–III NS II–IV NS III–IV NS
		Neither good nor bad (II)	36	3.52	1.72	3.60		
		Good (III)	39	4.02	1.61	4.40		
		Very good (IV)	7	5.03	0.73	5.20		
FSFI Pain	Education	Primary (I)	5	3.04	2.86	4.00	0.400	-
		Vocational (II)	21	4.78	1.75	6.00		
		Secondary (III)	47	4.18	2.25	5.20		
		Tertiary (IV)	20	4.56	2.15	6.00		
	Marital status	Single (I)	25	5.07	1.47	6.00	0.371	-
		Married (II)	32	4.25	1.92	4.60		
		Divorced (III)	19	3.58	2.87	5.60		
		Widowed (IV)	16	4.20	2.46	6.00		
	Relationship status	Formal (I)	32	4.28	1.93	4.60	0.772	-
		Informal (II)	21	4.57	2.30	5.60		
		None (III)	40	4.26	2.30	6.00		
	Financial situation	Bad (I)	11	4.73	2.41	6.00	0.340	-
		Neither good nor bad (II)	36	3.92	2.30	4.40		
		Good (III)	39	4.41	2.10	5.60		
		Very good (IV)	7	5.43	0.60	5.20		
Overall FSFI	Education	Primary (I)	5	15.52	11.29	19.20	0.080	-
		Vocational (II)	21	21.15	7.71	22.50		
		Secondary (III)	47	18.36	10.25	16.00		
		Tertiary (IV)	20	24.29	9.01	28.50		
	Marital status	Single (I)	25	21.04	10.30	22.00	0.245	-
		Married (II)	32	21.77	8.04	22.50		
		Divorced (III)	19	19.65	11.15	26.90		
		Widowed (IV)	16	15.43	9.63	12.60		
	Relationship status	Formal (I)	32	21.53	8.00	22.50	0.014*	I–II NS I–III NS II–III 0.014*
		Informal (II)	21	24.18	9.41	27.80		
		None (III)	40	16.84	10.26	13.20		
	Financial situation	Bad (I)	11	10.68	6.16	10.30	0.002*	I–II NS I–III 0.005* I–IV 0.004* II–III NS II–IV NS III–IV NS
		Neither good nor bad (II)	36	19.37	9.91	19.50		
		Good (III)	39	22.12	9.35	23.50		
		Very good (IV)	7	27.54	2.00	27.80		

FSFI, Female Sexual Function Index; IIEF-15, International Index of Erectile Function; M, mean; Me, median; NS - non significant; SD, standard deviation; K–W, Kruskal–Wallis test; p, *p*-value; *statistically significant value.

The analysis demonstrated that the level of formal education also significantly influenced certain domains of women’s sexual function (Table 5). Statistically significant differences were observed in the following areas: excitement (*p* = 0.029), where women with tertiary education achieved the highest scores, while those with primary and vocational education achieved the lowest scores. The difference was significant between women with secondary and tertiary education (*p* = 0.030), and lubrication (*p* = 0.016), and the highest scores were recorded by women with tertiary education, and the lowest by those with secondary and primary education; a significant difference was found between women with secondary and tertiary education (*p* = 0.013). In other FSFI domains, no statistically significant differences were found in relation to formal educational attainment.

Analysis of the IIEF-15 scores indicated that marital status did not have a significant relationship on most areas of sexual function. The domain of sexual desire was an exception, where the differences were statistically significant (*p* < 0.001). Unmarried men scored higher in this domain than married men (*p* = 0.012) and widowers (*p* = 0.002). The differences between divorced respondents and other groups were not statistically significant (Table 5).

Analysis of the FSFI scores revealed that sexual desire in women differed significantly depending on marital status (*p* < 0.001) (Table 5). The lowest scores were recorded among widows, who achieved significantly lower levels of sexual desire than single (*p* = 0.002) and divorced women (*p* < 0.001). Divorced women, in turn, achieved higher scores than married women (*p* = 0.002).

The analysis indicated that the relationship status of men (IIEF-15) did not significantly impact sexual function in any of the examined domains; differences between men in formal relationships, informal relationships, and those not in a relationship were not statistically significant.

In the case of women (FSFI), the relationship status had a significant impact on the following aspects of sexual function: desire—women in informal relationships scored higher than women in formal relationships (*p* = 0.011); arousal—women not in a relationship achieved significantly lower scores than women in formal relationships (*p* = 0.013) and informal relationships (*p* = 0.003); satisfaction—lower scores were reported by women not in a relationship compared to those in formal relationships (*p* = 0.031) and informal relationships (p = 0.002). Similarly, in the case of the overall FSFI score, women in informal relationships scored highest, differing significantly from women who were not in a relationship (*p* = 0.014) (Table 5).

The analysis revealed significant differences in erectile function (*p* = 0.044) and orgasmic function (*p* = 0.012) depending on financial status. Men in a bad financial situation scored significantly lower in both domains compared to those in a very good financial situation (*p* = 0.049 and *p* = 0.036, respectively) (Table 5).

#### 3.3.3 The level of formal education, relationship status, and financial situation and sexual quality of life

The analysis indicated a significant relationship of formal education level on sexual quality of life in both men and women (Table 6). The highest mean scores on the SQoL were achieved by those with tertiary education, in both male and female groups. The lowest scores were recorded among men with vocational education and women with secondary education. The differences between the groups were statistically significant; for men: *p* = 0.003 (significant *post hoc* difference between men with vocational education and tertiary education: *p* = 0.008); for women: *p* = 0.007 (significant post hoc difference between women with secondary education and tertiary education: *p* = 0.004). In the overall analysis, the differences were statistically significant (*p* < 0.001). Those with tertiary education achieved the highest sexual quality of life, significantly higher than those with vocational education (*p* = 0.002) and secondary education (*p* < 0.001).

**Table 6:** The level of formal education, relationship status and financial situation and sexual quality of life.

Scale	Variable		*n*	*M*	SD	Me	K-W	*p* *Post hoc*
SQoL-M	Education	Primary (I)	20	0.68	0.28	0.76	0.003*	I–II NS I–III NS I–IV NS II–III NS II–IV 0.008* III–IV NS
		Vocational (II)	45	0.56	0.24	0.65		
		Secondary (III)	18	0.67	0.29	0.75		
		Tertiary (IV)	13	0.77	0.20	0.87		
	Relationship status	Formal (I)	51	0.62	0.25	0.67	-	I–II NS I–III NS II–III NS
		Informal (II)	16	0.79	0.10	0.80		
		None (III)	31	0.59	0.31	0.67		
	Financial situation	Bad (I)	9	0.46	0.41	0.71	0.440	–
		Neither good nor bad (II)	34	0.64	0.22	0.67		
		Good (III)	51	0.66	0.25	0.75		
		Very good (IV)	4	0.78	0.09	0.75		
SQoL-F	Education	Primary (I)	5	0.67	0.16	0.65	0.007*	I–II NS I–III NS I–IV NS II–III NS II–IV NS III–IV 0.004*
		Vocational (II)	21	0.56	0.27	0.52		
		Secondary (III)	47	0.48	0.27	0.48		
		Tertiary (IV)	20	0.74	0.24	0.81		
	Relationship status	Formal (I)	32	0.65	0.20	0.62	-	I–II NS I–III NS II–III NS
		Informal (II)	21	0.62	0.35	0.81		
		None (III)	40	0.47	0.26	0.34		
	Financial situation	Bad (I)	11	0.48	0.25	0.31	0.003*	I–II NS I–III NS I–IV NS II–III 0.007* II–IV NS III–IV NS
		Neither good nor bad (II)	36	0.45	0.27	0.35		
		Good (III)	39	0.66	0.25	0.68		
		Very good (IV)	7	0.75	0.20	0.81		
Overall SQoL	Education	Primary (I)	25	0.68	0.26	0.75	<0.001*	–II NS I–III NS I–IV NS II–III NS II–IV 0.002* III–IV < 0.001*
		Vocational (II)	66	0.56	0.25	0.59		
		Secondary (III)	65	0.54	0.29	0.56		
		Tertiary (IV)	33	0.75	0.22	0.81		
	Relationship status	Formal (I)	83	0.64	0.23	0.67	0.001*	I–II NS I–III NS II–III 0.001*
		Informal (II)	37	0.69	0.28	0.81		
		None (III)	71	0.52	0.28	0.58		
	Financial situation	Bad (I)	20	0.47	0.32	0.48	<0.001*	I–II NS I–III 0.044* I–IV 0.028* II–III 0.019* II–IV 0.044* III–IV NS
		Neither good nor bad (II)	70	0.54	0.26	0.55		
		Good (III)	90	0.66	0.25	0.75		
		Very good (IV)	11	0.76	0.16	0.81		

M, mean; Me, median; SD, standard deviation; SQoL-F, Sexual Quality of Life, Female; SQoL-M, Sexual Quality of Life, Male; K–W, Kruskal–Wallis test; NS - non significant; p, *p*-value; *statistically significant value.

The analysis of sexual quality of life (SQoL) by status of relationship revealed significant differences among all respondents (*p* = 0.001). Those in informal relationships had significantly higher SQoL scores compared to those who were not in a relationship (*p* = 0.001) (Table 6).

In terms of sexual quality of life (SQoL) in women, those who rated their financial situation as “neither good nor bad” scored significantly lower than women with a good financial situation (*p* = 0.007). In the overall analysis, individuals in bad and average financial situations achieved significantly lower SQoL scores than those who reported good (*p* = 0.044 for I–III; *p* = 0.019 for II–III) and very good (*p* = 0.028 for I–IV; *p* = 0.044 for II–IV) financial situations (Table 6).

### 3.4 Correlations between IIEF and FSFI subscales and sexual quality of life

Among men, all domains of sexual function according to the IIEF-15 showed significant positive correlations with sexual quality of life (SQoL-M). The strongest correlations were found in overall satisfaction (*p* < 0.001), sexual desire (*p* < 0.001), erectile function (*p* < 0.001), and intercourse satisfaction (*p* < 0.001). Slightly weaker correlations were noted for orgasmic function (*p* = 0.006) (Table 7).

**Table 7:** Correlations between sexual function and sexual quality of life in men.

Scale	I	II	III	IV	V	SQoL-M
IIEF-15 Erectile Function (I)	1	*r* = 0.824 *p* < 0.001*	*r* = 0.703 *p* < 0.001*	*r* = 0.754 *p* < 0.001*	*r* = 0.630 *p* < 0.001*	*r* = 0.351 *p* < 0.001*
IIEF-15 Orgasmic Function (II)	*r* = 0.824 *p* < 0.001*	1	*r* = 0.654 *p* < 0.001*	*r* = 0.836 *p* < 0.001*	*r* = 0.671 *p* < 0.001*	*r* = 0.278 *p* = 0.006*
IIEF-15 Sexual Desire (III)	*r* = 0.703 *p* < 0.001*	*r* = 0.654 *p* < 0.001*	1	*r* = 0.630 *p* < 0.001*	*r* = 0.491 *p* < 0.001*	*r* = 0.445 *p* < 0.001*
IIEF-15 Intercourse satisfaction (IV)	*r* = 0.754 *p* < 0.001*	*r* = 0.836 *p* < 0.001*	*r* = 0.630 *p* < 0.001*	1	*r* = 0.736 *p* < 0.001*	*r* = 0.388 *p* < 0.001*
IIEF-15 Overall satisfaction (V)	*r* = 0.630 *p* < 0.001*	*r* = 0.671 *p* < 0.001*	*r* = 0.491 *p* < 0.001*	*r* = 0.736 *p* < 0.001*	1	*r* = 0.464 *p* < 0.001*
SQOL-M	*r* = 0.351 *p* < 0.001*	*r* = 0.278 *p* = 0.006*	*r* = 0.445 *p* < 0.001*	*r* = 0.388 *p* < 0.001*	*r* = 0.464 *p* < 0.001*	1

IIEF-15, International Index of Erectile Function; SQoL-M, Sexual Quality of Life, Male; r, Spearman’s rank correlation coefficient; p, *p*-value; *statistically significant value.

In women, all domains of sexual function, as measured by the FSFI, were strongly and statistically significantly correlated with the overall score on this scale (*p* < 0.001).

The strongest correlations were found between lubrication and orgasm (*p* < 0.001), lubrication and arousal (*p* < 0.001), and lubrication and satisfaction (*p* < 0.001). In terms of sexual quality of life (SQoL-F), significant positive correlations were observed with satisfaction (*p* < 0.001), lubrication (*p* < 0.001), arousal (*p* < 0.001), orgasm (*p* < 0.001), and overall FSFI score (*p* < 0.001) (Table 8).

**Table 8:** Correlations between sexual function and sexual quality of life in women.

Scale	I	II	III	IV	V	VI	FSFI	SQOL-F
FSFI Desire (I)	1	*r* = 0.230 *p* = 0.026*	*r* = 0.283 *p* = 0.006*	*r* = 0.286 *p* = 0.006*	*r* = 0.320 *p* = 0.002*	*r* = 0.085 *p* = 0.418	*r* = 0.412 *p* < 0.001*	*r* = 0.010 *p* = 0.921
FSFI Arousal (II)	*r* = 0.230 *p* = 0.026*	1	*r* = 0.878 *p* < 0.001*	*r* = 0.779 *p* < 0.001*	*r* = 0.805 *p* < 0.001*	*r* = 0.393 *p* < 0.001*	*r* = 0.900 *p* < 0.001*	*r* = 0.514 *p* < 0.001*
FSFI Lubrication (III)	*r* = 0.283 *p* = 0.006*	*r* = 0.878 *p* < 0.001*	1	*r* = 0.891 *p* < 0.001*	*r* = 0.865 *p* < 0.001*	*r* = 0.465 *p* < 0.001*	*r* = 0.944 *p* < 0.001*	*r* = 0.552 *p* < 0.001*
FSFI Orgasm (IV)	*r* = 0.286 *p* = 0.006*	*r* = 0.779 *p* < 0.001*	*r* = 0.891 *p* < 0.001*	1	*r* = 0.906 *p* < 0.001*	*r* = 0.379 *p* < 0.001*	*r* = 0.907 *p* < 0.001*	*r* = 0.510 *p* < 0.001*
FSFI Satisfaction (V)	*r* = 0.320 *p* = 0.002*	*r* = 0.805 *p* < 0.001*	*r* = 0.865 *p* < 0.001*	*r* = 0.906 *p* < 0.001*	1	*r* = 0.351 *p* < 0.001*	*r* = 0.907 *p* < 0.001*	*r* = 0.585 *p* < 0.001*
FSFI Pain (VI)	*r* = 0.085 *p* = 0.418	*r* = 0.393 *p* < 0.001*	*r* = 0.465 *p* < 0.001*	*r* = 0.379 *p* < 0.001*	*r* = 0.351 *p* < 0.001*	1	*r* = 0.552 *p* < 0.001*	*r* = 0.199 *p* = 0.056
Overall FSFI	*r* = 0.412 *p* < 0.001*	*r* = 0.900 *p* < 0.001*	*r* = 0.944 *p* < 0.001*	*r* = 0.907 *p* < 0.001*	*r* = 0.907 *p* < 0.001*	*r* = 0.552 *p* < 0.001*	1	*r* = 0.536 *p* < 0.001*
SQOL-F	*r* = 0.010 *p* = 0.921	*r* = 0.514 *p* < 0.001*	*r* = 0.552 *p* < 0.001*	*r* = 0.510 *p* < 0.001*	*r* = 0.585 *p* < 0.001*	*r* = 0.199 *p* = 0.056	*r* = 0.536 *p* < 0.001*	1

FSFI, Female Sexual Function Index; SQoL-F, Sexual Quality of Life, Female; r, Spearman’s rank correlation coefficient; p, *p*-value; *statistically significant value.

## 4 Discussion

The results of this study revealed significant differences in sexual function between people living with HIV and lung cancer patients. According to the data obtained, men living with HIV achieved significantly higher erectile (*p* = 0.011) and desire (*p* < 0.001) scores compared to cancer patients. This finding is consistent with available data indicating that cancer, including lung cancer, has a significant negative impact on sexual function. A meta-analysis of 43 studies involving a total of 13,148 cancer patients with various tumor sites, conducted by Pizzol et al., found that sexual dysfunction is common among patients with cancer, including lung cancer. The mean prevalence of erectile dysfunction in cancer patients was 40.72% (Pizzol et al., 2021). Bolat et al., who studied 50 patients with lung cancer to investigate the impact of treatment of lung cancer on sexual function, also confirmed that patients with lung cancer had significantly worse sexual function (Bolat et al., 2019). It found that the main cause of this dysfunction was fear of death (Costa et al., 2025). The deterioration of sexual function is not limited to physiological aspects but also includes psychological components such as diminished sense of attractiveness, fear of rejection, and avoidance of intimacy (Florez et al., 2024).

In the group of patients living with HIV, higher scores were observed for sexual desire and sexual functioning compared with individuals diagnosed with lung cancer. These differences were statistically significant in selected domains, particularly those related to sexual desire (*p* < 0.001) and erectile function (*p* = 0.020), to the disadvantage of patients with lung cancer. Overall sexual quality of life was comparable between the groups, with slightly higher values among men living with HIV (0.66 vs. 0.62) and, conversely, lower values among women with HIV compared to those with lung cancer (0.62 vs. 0.50). These results partially correlate with observations from the Sexual Health Assessment in Women with Lung Cancer (SHAWL) study, which included 249 women with lung cancer, in which 77% of participants reported very low interest in or no interest in sex, and 48% expressed dissatisfaction with their sexual life (Duma et al., 2022). Another study, conducted among patients enrolled in the CLARIFY H2020 project (2020–2022), which evaluated the frequency and quality of sexual activity among cancer patients (a total of 383 patients, including 101 with lung cancer), found sexual dissatisfaction among 76% of women and 24% of men. Women with lung cancer reported significant arousal dysfunction (*p* = 0.016) and overall sexual dissatisfaction (*p* = 0.044) (Ospina-Serrano et al., 2024). In the study by Siegel et al., which included 284 women living with HIV, psychological factors such as anxiety, low self-esteem, and fear of rejection were found to significantly influence the subjective assessment of sexual life among these women. These mechanisms may explain the observed discrepancy between heightened sexual desire and overall sexual quality of life (Siegel et al., 2006). Similarly to the present study, women living with HIV often rated their sexual quality of life lower than did female oncology patients.

Our study clearly demonstrates that women with lung cancer—despite physical limitations—can maintain a relatively high sexual quality of life thanks to emotional closeness and support from their partners. Similar findings emerged from a study by Reese et al. (2022). They showed that emotional relationships positively influence sexual satisfaction, despite the significant physical limitations caused by the disease and treatment, which lead to serious, long-term sexual challenges (e.g., loss of interest in sex, vaginal dryness, and discomfort). Furthermore, it was demonstrated that perceiving the relationship as having an “expiration date” affects the attitudes of patients and their partners toward sexuality and intimacy, and also makes it more challenging to cope with these difficulties (Reese et al., 2022).

When analyzing the relationship of age on sexual function, significant negative correlations between age and most IIEF-15 domains were observed in men, both in people living with HIV and in lung cancer patients (erection, orgasm, desire, sexual satisfaction), suggesting that older age is associated with reduced sexual function. This finding is consistent with common clinical and epidemiological knowledge, which indicates that age is a strong risk factor for sexual dysfunction. Similar findings were reported in a prospective 12-year study of 625 participants (54.4% HIV-negative, 45.6% HIV-positive) by Mustapha et al., who found that men with HIV had a 41% higher risk of erectile dysfunction, which increased by 19% with each subsequent five-year period of life (Mustapha et al., 2024). Our study in a group of women showed a negative correlation between age and desire, while a surprising finding was a positive correlation between age and sexual quality of life (*p* = 0.022). This may indicate greater adaptability in older women or a shift in emphasis from physiological functions to emotional and relational aspects of sexual life. Some studies suggest that women adapt better to chronic illness and are able to redefine their sexuality and relationship quality despite health limitations (Manninen et al., 2022). Williams et al. studied lung cancer patients and found that older adults expressed their sexuality through closeness, intimacy, and non-sexual behavior in the traditional sense (not related to intercourse). Emotional intimacy and support from a stable partner played a key role in helping patients adapt to physical limitations (Williams et al., 2013).

An analysis of the correlation between sexual function and sexual quality of life revealed significant relationships in both men and women, with some differences in the strength and nature of these relationships. In men, all domains of sexual function examined (IIEF-15) were strongly correlated (*p* < 0.001). The strongest correlations were observed between erectile function, orgasm, and intercourse satisfaction, indicating the consistency of these aspects of sexual health in men. Sexual quality of life (SQoL-M) was moderately correlated with individual dimensions of sexual function, particularly sexual desire (*p* < 0.001) and intercourse satisfaction (*p* < 0.001). Positive correlations between all FSFI/IIEF-15 domains and SQoL suggest that a decline in any of the functions (e.g., erection, lubrication, orgasm) negatively affects the overall perception of intimate life.

This phenomenon was also described in a meta-analysis conducted on 13,148 patients with cancer, performed by Pizzol et al. (2021). The results showed that the mean prevalence of erectile dysfunction in this population was 40.72%. Erectile dysfunction was significantly correlated with the anticancer treatment used, the location of the tumor, and the age of the patients. Such a high incidence of erectile dysfunction among men with cancer indicates a significant impact of the disease and its treatment on sexual function. Given that sexual function is one of the key components of sexual quality of life, these findings highlight the need for comprehensive care for cancer patients, including sexual health (Pizzol et al., 2021). These correlations may also confirm that subjective feelings about the sexual quality of life depend not only on physical aspects of sexual function but also on emotional satisfaction.

Similar conclusions were reached by Laumann et al., who studied a group of 1,749 women and 1,410 men aged 18–59, showing that sexual disorders were more common in individuals with emotional problems, which led the researchers to conclude that emotional issues may contribute to these disorders. The study highlights that sexual quality of life depends not only on physical aspects but also on emotional health and relationship satisfaction, especially in women (Laumann et al., 1999).

Our study revealed significant differences in all domains of the FSFI and SQoL-F among women, depending on their relationship status, with the highest scores observed in women in informal relationships and the lowest scores in single women. These findings confirm the role of partnerships as a protective factor for sexual health, both physically and emotionally. In men, marital status had a significant relationship only on sexual desire, with higher scores reported by single men. These differences may stem from different biological and psychological mechanisms influencing the sexuality of both sexes.

Similar conclusions can also be drawn from Laumann et al. (1999) and Clayton et al. (2006), who indicated that physical factors related to erectile function and orgasm play a significant role in men, while sexuality is more holistic in women and strongly linked to the emotional and psychological aspects of relationships. A qualitative study conducted on lung cancer patients by Lindau et al. indicated that older patients (≥65 years) adapt better emotionally, build stronger relationships, and communicate better, which translates into better sexual function despite physical limitations (Lindau et al., 2011).

Formal educational level also had a significant relationship on the variables included in our study. Men with vocational education had the lowest scores for erection and sexual desire, while men with tertiary education achieved significantly better scores in all domains of the IIEF-15 and SQoL-M. Similarly, women with tertiary education scored higher on arousal, lubrication, and quality of sexual life. This can be attributed to increased health and sexual awareness, easier access to treatment, reduced social stigma, improved access to health information, a better understanding of the treatment process, and a greater ability to cope with stress, which can lead to a better quality of life, including a better sexual quality of life.

The literature indicates that patients with tertiary education are more likely to use pharmacological and psychological therapies and sexual counseling, which translates into better clinical outcomes. This relationship was also described in a study that analyzed 18 systematic reviews of studies on factors affecting the quality of life of cancer patients, conducted between 2012 and 2023 by Marzorati et al. (2025). The study identified several psychosocial factors that affect the quality of life of cancer patients. One of these was formal educational level, which translated significantly into patients’ ability to cope with their disease and its treatment. Although this study did not focus specifically on the sexual quality of life, it found that the level of formal education indirectly impacted this area of patients’ lives (Marzorati et al., 2025).

In the LUDICAS study, it was also demonstrated that a higher level of formal education acted as a protective factor against severe sexual dysfunction in patients with lung cancer—individuals with higher education had approximately a 71% lower risk of experiencing severe sexual dysfunction compared with those with lower educational attainment (Ospina-Serrano et al., 2025).

In our study, financial situation turned out to be a significant predictor of sexual quality of life. Both men and women who reported bad financial situations had the lowest scores for sexual function and sexual satisfaction. A low financial status may not only limit access to appropriate treatment but also contribute to reduced self-esteem and attractiveness, which can negatively relationship intimate life. Similar relationships were observed by Peyre et al. (2019), who studied 690 individuals living with HIV/AIDS in France. The authors showed that lower satisfaction with sexual life was significantly associated with unemployment, low income, and low self-esteem. These findings confirm that socio-economic factors are key determinants of sexual quality of life, regardless of the underlying disease (Peyre et al., 2019). Our analysis also confirms the findings reported by Forrest et al. (2013), who demonstrated in their meta-analysis that patients with lower socio-economic status were less likely to receive oncological treatment for lung cancer. Importantly, these inequalities persisted regardless of the stage of the disease and the type of healthcare system (public/private), indicating that they are not merely the result of later diagnosis or limited access to healthcare services. This means that in addition to clinical and medical factors, economic barriers also affect access to treatment. Understanding and addressing these barriers is key to reducing treatment inequalities and improving quality of life and prognosis in lung cancer patients (Forrest et al., 2013).

The analysis shows that sexual health should be an integral part of caring for patients with chronic diseases, both cancer and infectious ones. Particular attention should be paid to older adults, single people, those with lower levels of education and those in difficult economic circumstances, as they are most likely to experience sexual problems and a deterioration in their quality of life in this area.

In everyday clinical practice, it is necessary to implement measures including couples counseling and sex education. It is also important to talk about sexuality, regardless of age, sex, or the type of chronic disease the patient lives with.

The comparison of different patient groups proved to be a practical research approach, allowing us to identify both common mechanisms influencing sexual satisfaction in chronic diseases and problems specific to particular diseases.

### 4.1 Research limitations

One of the significant limitations of our study is the failure to take into account certain factors that may affect sexual function, such as chronic fatigue, pain, shortness of breath, symptoms of depression, or the quality of the relationship. An additional limitation is the relatively small size of the study group and the fact that all participants came from a single center—the University Teaching Hospital in Bialystok—which means that the study covered a population from a limited geo-graphical area. Failure to consider the above factors may result in an inability to interpret our results in greater depth. Future research should consider including the afore-mentioned factors in the analysis and conducting studies on a larger and more diverse population.

## 5 Conclusion

The conducted study confirmed that sociodemographic factors play a crucial role in shaping the sexual functioning of patients living with HIV and those diagnosed with lung cancer. Age, formal education, financial situation, marital status, and status of relationship were significantly associated with both the level of sexual functioning and the subjective assessment of sexual quality of life. Patients living with HIV demonstrated better outcomes in terms of sexual functioning and sexual quality of life compared with those with lung cancer, which may indicate greater psychological adaptation and better preservation of sexual function despite the chronic nature of the disease.

Moreover, higher education, stable partnerships, and a more favorable financial situation were associated with higher scores on scales assessing sexual functioning (IIEF-15, FSFI) and sexual quality of life (SQoL). The observed relationships between all domains of sexual functioning and sexual quality of life emphasize the need for a holistic approach to the care of chronically ill patients—one that integrates emotional, social, and relational aspects. Such an approach may contribute to improving both overall quality of life and sexual satisfaction in this patient population.

Future studies should expand the analysis to include additional factors such as pain, chronic fatigue, and depressive symptoms, as well as involve a larger patient cohort to enable a more comprehensive assessment of the determinants of sexual functioning in the course of chronic diseases.

## Data Availability

The raw data supporting the conclusions of this article will be made available by the authors, without undue reservation.
